# Aldehyde Dehydrogenase (ALDH) Activity Does Not Select for Cells with Enhanced Aggressive Properties in Malignant Melanoma

**DOI:** 10.1371/journal.pone.0010731

**Published:** 2010-05-20

**Authors:** Lina Prasmickaite, Birgit Ø. Engesæter, Nirma Skrbo, Tina Hellenes, Alexandr Kristian, Nina K. Oliver, Zhenhe Suo, Gunhild M. Mælandsmo

**Affiliations:** 1 Department of Tumour Biology, Oslo University Hospital Radiumhospitalet, Oslo, Norway; 2 Department of Surgery, Oslo University Hospital Radiumhospitalet, Oslo, Norway; 3 Department of Pathology, Oslo University Hospital Radiumhospitalet, Oslo, Norway; 4 Department of Pharmacy, Faculty of Health Sciences, University of Tromsø, Tromsø, Norway; Emory Unviersity, United States of America

## Abstract

**Background:**

Malignant melanoma is an exceptionally aggressive, drug-resistant and heterogeneous cancer. Recently it has been shown that melanoma cells with high clonogenic and tumourigenic abilities are common, but markers distinguishing such cells from cells lacking these abilities have not been identified. There is therefore no definite evidence that an exclusive cell subpopulation, i.e. cancer stem cells (CSC), exists in malignant melanoma. Rather, it is suggested that multiple cell populations are implicated in initiation and progression of the disease, making it of importance to identify subpopulations with elevated aggressive properties.

**Methods and Findings:**

In several other cancer forms, Aldehyde Dehydrogenase (ALDH), which plays a role in stem cell biology and resistance, is a valuable functional marker for identification of cells that show enhanced aggressiveness and drug-resistance. Furthermore, the presence of ALDH^+^ cells is linked to poor clinical prognosis in these cancers. By analyzing cell cultures, xenografts and patient biopsies, we showed that aggressive melanoma harboured a large, distinguishable ALDH^+^ subpopulation. *In vivo*, ALDH^+^ cells gave rise to ALDH^−^ cells, while the opposite conversion was rare, indicating a higher abilities of ALDH^+^ cells to reestablish tumour heterogeneity with respect to the ALDH phenotype. However, both ALDH^+^ and ALDH^−^ cells demonstrated similarly high abilities for clone formation *in vitro* and tumour initiation *in vivo*. Furthermore, both subpopulations showed similar sensitivity to the anti-melanoma drugs, dacarbazine and lexatumumab.

**Conclusions:**

These findings suggest that ALDH does not distinguish tumour-initiating and/or therapy-resistant cells, implying that the ALDH phenotype is not associated with more-aggressive subpopulations in malignant melanoma, and arguing against ALDH as a “universal” marker. Besides, it was shown that the ability to reestablish tumour heterogeneity is not necessarily linked to the more aggressive phenotype.

## Introduction

Malignant melanoma is an exceptionally aggressive type of human cancer, known for its high metastatic potential and notorious resistance against all major chemotherapeutic drugs. The prognosis for metastatic melanoma patients is very poor: a median survival of stage IV disease is only ∼6 months with only 5% surviving 5 years [1], [2]. Despite large efforts during the last decades testing various treatment strategies, none significantly prolonged patient survival [3], indicating that melanoma cells possess efficient mechanism for developing resistance to therapy. Recently it has been hypothesised that tumour initiation as well as therapy resistance might be associated to the presence of cells with stem cell properties, so called cancer stem cells (CSC) [4]. However, in malignant melanoma conflicting results have been reported regarding the existence of such distinct CSC subpopulations [5]–[7]. The recent study by Quintana et al. [7] revealed that none of the tested surface markers for CSCs, identifying tumour-initiating stem-like cells in other cancers, could distinguish between tumourigenic and non-tumourigenic melanoma cells. Furthermore, the same study has shown that a large fraction, at least 25% of random single cells isolated from melanoma patient biopsies, had tumourigenic potential [7]. In agreement with this, recently we have shown that 20–60% of randomly chosen (i.e. regardless CSC marker expression) single melanoma cells, isolated from cell cultures and xenografts, were highly clonogenic and self-renewing [8]. Altogether, this opposes the CSC hypothesis claiming that cells with tumourigenic properties are rare and distinguishable from the tumour cells lacking such properties. Also, this suggests that if the “markers” for discriminating cells with enhanced tumourigenic potential will be identified in melanoma in the future, they will most likely mark a relatively large cell subpopulation.

Lately, Aldehyde Dehydrogenase (ALDH), particularly its isoform 1 has received considerable attention as a functional marker for identification of cells with enhanced tumourigenic/metastatic potential and elevated therapeutic resistance in several cancers of epithelial origin [9]–[12]. ALDH is a detoxifying enzyme responsible for the oxidation of intracellular aldehydes, thereby mediating self-protection and resistance to some alkylating agents used in cancer therapy [13]. Besides, ALDH is implicated in the biology of normal stem cells through its role in metabolism of retinol to retinoic acid, which initiates a program of cellular differentiation [14]. Therefore, ALDH has been suggested as a marker for isolating normal stem cells and lately also CSCs from several tumour types (reviewed in [15]). Importantly, it has been reported that the presence of cells with ALDH activity correlated with poor clinical prognosis in breast and lung cancers [10], [11], [16]. However, in ovarian carcinoma, ALDH has been found to be a favorable prognostic factor [17], suggesting that ALDH functions as a ”marker” of aggressive tumour cells in some contexts, but not in others.

In malignant melanoma, the association between a biologically aggressive phenotype and the presence of ALDH^+^ cells has not been studied. The aim of the present study was to investigate the role of melanoma cells with ALDH activity for tumourigenicity and therapeutic resistance. Our data indicate that despite the presence of a large, clearly distinguishable subpopulation of ALDH^+^ cells, they did not demonstrate higher biological aggressiveness compared to ALDH^−^ cells. The latter observation argues against ALDH as a marker for distinguishing tumour-initiating and/or therapy-resistant cells in malignant melanoma. ALDH^+^ cells, therefore, may play a different role in melanoma than in other cancers like epithelial cancers. In the absence of clarified markers that can distinguish tumourigenic and therapy resistant from nontumourigenic and sensitive cells, there is so far lack of evidence that malignant melanoma is hierarchically organized and follows a CSC model.

## Materials and Methods

### Ethics statement

Collection and the use of biopsies from metastatic melanoma patients were approved by the South-East National Committee for Medical and Health Research Ethics (REK, approval no: S-01252 and 2.2007.997) and by the institutional data protection official at Oslo University Hospital. The written informed consent was obtained from all patients involved in the study.

### Metastatic melanoma models: cell cultures and xenografts

To isolate tumour cells, patient biopsies were mechanically disintegrated in cold PBS supplemented with 0.4% human serum albumin. The melanoma cells were separated by magnetic beads conjugated to the 9.2.27 antibody (9.2.27Ab) [18] (kindly provided by Dr. R.Reisfeld, La Jolla, CA), which binds to the High Molecular Weight-Melanoma-Associated Antigen (HMW-MAA) expressed on tumour cells. Metastatic melanoma low-passage cultures, Melmet 1 and Melmet 5, were established from subcutaneous and lymph node (LN) metastases, respectively as described previously [8]. Briefly, the isolated melanoma cells were grown as traditional monolayers (MON) in RPMI medium supplemented with 10% foetal calf serum (FCS) and 2 mM Glutamax (both BioWittaker, Belgium) or as non-adherent spheroids (SPH) in serum-free human embryonic stem cell medium (hESCM) as described previously [5] in a 5% CO_2_ atmosphere at 37°C.

Melmet xenografts were established in nude mice by subcutaneous (s.c) injection of 50,000–250,000 cells derived from the monolayer and the spheroid cultures of passages below 12.

### Aldefluor assay and identification of cells with enhanced ALDH activity

The Aldefluor kit (Stem Cell Technologies, Vancouver, BC, Canada) was used to identify cell populations with high ALDH enzymatic activity. Briefly, 10^6^ cells harvested from cell cultures, mechanically disintegrated xenografts or patient biopsies were resuspended in Aldefluor assay buffer containing ALDH substrate as recommended by the producer. As a negative control for all samples, an aliquot of “Aldefluor-exposed” cells was immediately quenched with a specific ALDH inhibitor, diethylaminobenzaldehyde (DEAB). Following 30 min incubation at 37°C, the cells were centrifuged and processed as follows: a) cells derived from the xenografts were resuspended in 100 µl Aldefluor buffer for the subsequent staining with the APC-labelled TRA-1-85 antibody (clone TRA-1-85, R&D Systems), which recognises human cells and thereby allows their discrimination from mouse cells; b) cells derived from patient biopsies were stained with the APC-labelled 9.2.27Ab, allowing identification of HMW-MAA expressing tumour cells. After incubation for 30 min at 4°C and following centrifugation, the cells were resuspended in cold Aldefluor buffer, stained with 1 µg/ml propidium iodide (PI) (Sigma) to discriminate viable cells from dead cells during the following analysis on LSRII or sorting on FACS DIVA flow cytometer (both from Beckton Dickinson). Aldefluor staining was detected in a green fluorescence channel FL1, and the samples treated with the inhibitor DEAB (+DEAB) were used as controls to set the gates defining the ALDH^+^ region. The gating strategy is presented in Figure S1. FlowJo 7.2.5 software was used to analyze the data.

### Evaluation of clonogenicity of single melanoma cells

ALDH^+^ and ALDH^−^ cells were isolated from Melmet 1 and Melmet 5 xenografts by FACS, distributing one viable cell per well in hESCM into 96-well plate. The presence of single cells was confirmed visually inspecting each well by a microscope shortly after FACS. Each well was supplemented with fresh hESCM every second day. After 3 weeks in culture, a number of wells containing expanded clones (spheroids) was counted manually. To confirm unlimited potential for self-renewal, the clones were disintegrated by EDTA into single cells, which were replated at a clonal density (1000 cells/ml hESCM) for evaluation of daughter spheroid formation. Eventually, the cells constituting daughter spheroids were reanalyzed by the Aldefluor assay.

### Evaluation of tumourigenicity *in vivo*

Following FACS-isolation of ALDH^+^ and ALDH^−^ subpopulations, an aliquot of the cell suspension was stained with trypan blue to discriminate dead cells from viable cells, which were counted using hemocytometer. The cells were resuspended at desired concentrations in serum-free RPMI medium supplemented with 2 mM Glutamax and 20 mM Hepes before injection 100 µl s.c. into NOD-SCID Il2rg^−/−^ mice (strain NOD.Cg-*Prkdc^scid^ Il2rg^tm1Wjl^*/SzJ, 5–8 weeks of age).

Tumour formation was observed for up to 6 months measuring tumour size weekly by a calliper. Tumour volume V was calculated as follows: V = W^2^×L×0.5, where W and L is tumour width and length, respectively. All procedures and experiments involving animals were approved by the National Animal Research Authority and were conducted according to the regulations of the Federation of European Laboratory Animals Science Association.

### Immunohistochemical staining

Formalin-fixed paraffin-embedded sections of tumour tissue from patient biopsies were subjected to immunohistochemical staining with ALDH1A1 antibody (rabbit polyclonal, clone ab51028, Abcam). In brief, after initial deparaffinization/hydration and antigen retrieval, the slides were incubated with the ALDH1A1 antibody at dilution 1∶300 in low pH buffer (Dako) for 1 h followed by incubation with a secondary antibody and eventually visualization by using DakoCytomation EnVision+ System-HRP. Tumour tissue sections stained only with the secondary antibody were used as negative controls, while human liver sections stained with the primary followed by the secondary antibody were used as a positive control.

### Detection of TRAIL-R2 level by flow cytometry

The Melmet cells (∼500,000) from monolayers were resuspended in 100 µl cold staining buffer (PBS containing 0.5% FCS and 3% human immune globulin gammagard (N.V Baxter S.A, Belgium) and stained with the primary antibody, mouse anti-TRAIL-R2 (clone DJR2–4 (a.k.a.7–8), eBioscience) for 30 min at 4°C followed by staining with the secondary antibody, AlexaFluor 647-labeled goat anti-mouse (Invitrogen). The stained samples and IgG isotype control samples were analyzed on LSRII flow cytometer, and the data were analyzed by FlowJo software.

### Therapy-related studies

For *in vitro* treatment of cells derived from the monolayer cultures, 3,000 and 150,000 cells (comprising both ALDH^+^ and ALDH^−^ subpopulations; the approximate percentage of each population before treatment is indicated in Figure S2A, B) were seeded into a well of 96-well and 6-well plates, respectively, and the next day treated with 100 µg/ml dacarbazine (DTIC) (Medac, Hamburg, Germany) or 10 µg/ml TRAIL-R2 agonist antibody lexatumumab (formerly HGS-ETR2; provided by Human Genome Sciences, Rockville, MD). Two days later, the 96-well plates were analyzed by CellTiter 96 Aqueous One solution, i.e. the MTS assay (Promega, Madison, WI) to evaluate cell viability, while the surviving cells from the 6-well plates were collected and analyzed by the Aldefluor assay to identify the percentage of ALDH^+^ and ALDH^−^ subpopulations after treatment. Untreated cells and cells exposed to non-specific IgG (provided by Human Genome Sciences) were used as controls and analyzed in parallel with the treated samples.

To evaluate the treatment effect selectively on ALDH^+^ and ALDH^−^ cells, the two subpopulations were FACS-isolated from the monolayer cultures and the xenografts, resuspended in RPMI medium and seeded into a 96-well plate for the MTS assay (3500–5000 cells/well) or 6-well pate for the clonogenic-assay (500 cells/well). Two days later, the cells were exposed to DTIC or lexatumumab for two days (for evaluation of a short-term effect by the MTS assay) or for two weeks (for evaluation of a long-term effect by the clonogenic assay, where colonies were defined when they contained >50 cells).

For *in vivo* treatment with DTIC, mice bearing Melmet tumours (30–50 mm^3^ in volume) were injected intraperitonally (i.p.) with 250 mg/kg DTIC. Three and five days later, the tumours were harvested and analyzed by the Aldefluor assay.

### Statistical analysis

To assess the statistical significance between ALDH^+^ and ALDH^−^ groups, two-tailed Student's t-test was done. Results were considered statistically significant if p<0.05.

## Results

### Identification of ALDH-positive cells in metastatic melanoma

Analysis of the Melmet 1 and Melmet 5 cultures (monolayers and spheroids), the corresponding xenografts and melanoma patient biopsies revealed the presence of ALDH^+^ tumour cells. All investigated Melmet cultures and xenografts harboured relatively large ALDH^+^ subpopulations, as illustrated by representative dot-plots in Figure S2A,B. Although, the percentage of ALDH^+^ cells varied among the different models and specific samples, it was observed that Melmet 1-based models usually had a larger fraction of ALDH^+^ cells (40–90%) than Melmet 5 models (8–20%). Also the xenografts derived from directly implanted clinical biopsies, i.e. omitting an *in vitro* culturing step, showed a large ALDH^+^ subpopulation (∼70%), (Figure S2C), confirming that the presence of ALDH^+^ cells in Melmet models is not a result of *in vitro* culturing.

**Figure 1:**
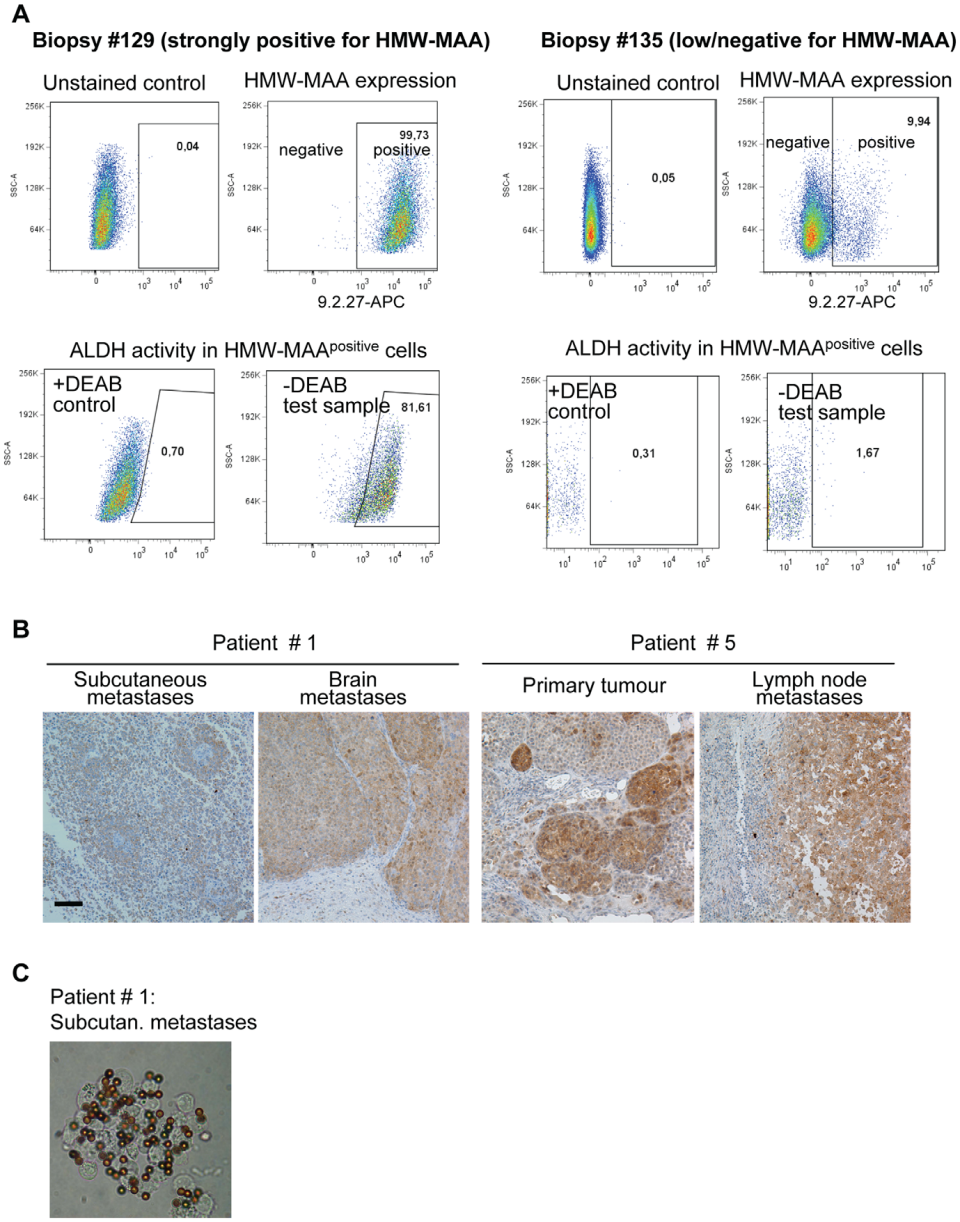
Identification of ALDH^+^ cells in patient biopsies. (A) Flow cytometric analysis of cells from fresh LN biopsies stained by Aldefluor and co-stained with APC-labelled 9.2.27Ab binding to HMW-MAA. Dot-plots show HMW-MAA staining (upper panel) and ALDH activity (lower panel) in two representative biopsies #129 and #135, differing in the HMW-MAA levels and the ALDH activity. “Unstained controls” were used to set the gates defining HMW-MAA^+^ cells; “+DEAB controls” were used to set the gates defining ALDH^+^ subpopulations in “−DEAB test samples”. A supplementary information regarding the analysis of these biopsies is presented in Figure S3. (B) ALDH immunostaining in the biopsies from patients #1 and #5: subcutaneous and brain metastases (from #1); primary tumour and LN metastases (from #5). Bar, 100 µm. (C) A representative picture, here for the biopsy from patient #1 (used for establishment of Melmet 1), illustrating 9.2.27Ab-magnetic beads binding to melanoma cells, which confirms HMW-MAA positivity in the biopsy.

Cells with ALDH activity were also identified in patient biopsies. The LN biopsies like #129, which contained melanoma cells with high expression of HMW-MAA, harboured a large ALDH^+^ subpopulation in the HMW-MAA^positive^ fraction (Figures 1A and S3). The biopsies like #135, which contained melanoma cells with significantly lower expression of HMW-MAA, had very few ALDH^+^ cells in the HMW-MAA^positive^ fraction (Figures 1A and S3). The same trend was observed in several other investigated biopsies from lymph nodes of melanoma patients (Figure S4). The HMW-MAA^negative^ fraction consisted mainly of non-melanoma cells (“normal” stromal cells), since there were hardly any cells expressing a melanocytic marker Melan A in this fraction (Figure S3). The HMW-MAA^negative^ cells did not show a notable ALDH actvity (Figures S3 and S4). This indicates that only HMW-MAA^high^ cells demonstrated high ALDH activity in the investigated biopsies. To note, it has been shown by others that HMW-MAA is related to a malignant potential in aggressive melanoma [19].

Immunohistochemical analysis of archived clinical material revealed ALDH expression in the biopsies from patients #1 and #5. Thus ALDH1A1 was identified in the subcutaneous and the LN metastases that were used for establishment of Melmet 1 and Melmet 5 cultures, respectively. Furthermore, ALDH1A1 was expressed also in the distant brain metastases and the primary tumour taken from patients #1 and #5, respectively (Figure 1B). The investigated biopsies from patient #1 and #5 were also strongly positive for HMW-MAA as detected by the efficient binding of 9.2.27Ab-magnetic beads as illustrated in Figure 1C. All this confirms that aggressive melanoma clearly positive for HMW-MAA also harbours ALDH^+^ cells.

### ALDH^+^ and ALDH^−^ cells are highly clonogenic *in vitro*

**Figure 2:**
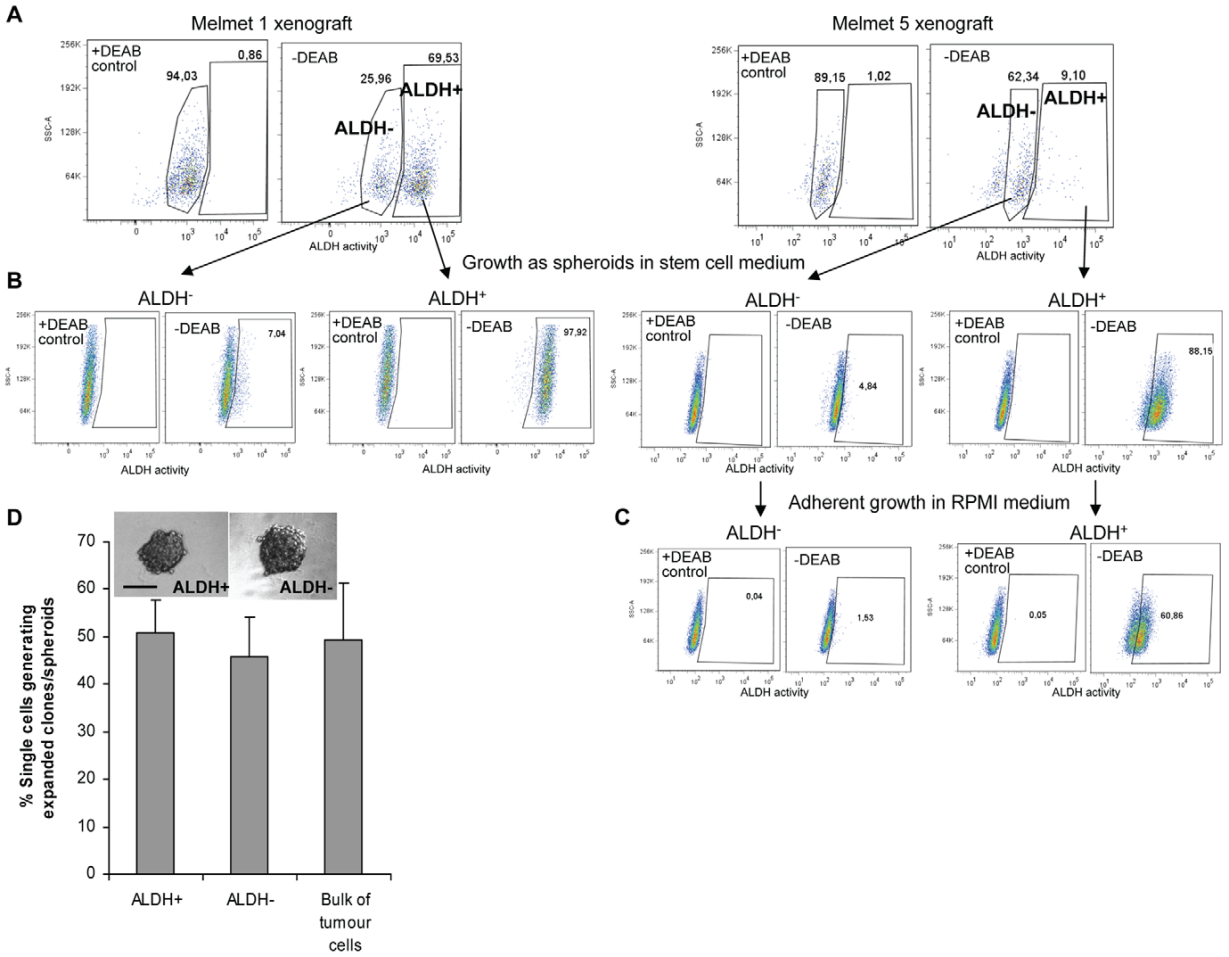
*In vitro* comparison of clonogenic potential of ALDH^+^ and ALDH^−^ cells isolated from melanoma xenografts. (A) ALDH^+^ and ALDH^−^ subpopulations identified in Melmet 1 and Melmet 5 xenografts. (B) Evaluation of the ALDH phenotype in the daughter spheroids formed during culturing of sorted ALDH^+^ and ALDH^−^ cells in hESCM as described in (D). (C) Aldefluor analysis of the spheroid-derived cells subsequently cultured adherently in RPMI for 2 weeks (representative dot-plots only for Melmet 5). (D) Sorted ALDH^+^ and ALDH^−^ cells were seeded one cell/well and cultured in hESCM to allow formation of single-cell-derived clones, spheroids showed in the insert (bar, 100 µm). Efficiency of spheroid formation from unsorted bulk melanoma cells is presented for comparison. Data represents mean ± SEM (n = 7). The formed spheroids from each group were collected, disintegrated into single cells that were further cultured in hESCM for formation of daughter spheroids. The latter were reanalyzed by the Aldefluor assay as shown in (B) or cultured further in RPMI as shown in (C).

To compare the clonogenic abilities, ALDH^+^ and ALDH^−^ melanoma cells were FACS-isolated from the Melmet 1 and the Melmet 5 xenografts, which contain a large and a considerably smaller ALDH^+^ subpopulation, respectively (Figure 2A). The sorted single cells were cultured in hESCM one cell/well in 96-well plates, and the clonogenic potential was evaluated by counting expanded clones, spheroids (illustrated in the Figure 2D insert). As shown in Figure 2D, a similar efficiency in spheroid formation and growth rate was observed for the single cells from both subpopulations. To evaluate the self-renewal capability, the formed spheroids were disintegrated into single cells and further cultured at a clonal density in hESCM. Efficient formation of daughter spheroids was observed for both ALDH^+^ and ALDH^−^ groups (data not shown). Further analysis by the Aldefluor assay revealed that the majority of the cells constituting the daughter spheroids kept the ALDH phenotype of the parental cell, i.e. either ALDH^+^ or ALDH^−^ (Figure 2B). This confirms that both subpopulations had clonogenic abilities and could repopulate themselves when cultured in hESCM for one month. When the disintegrated spheroid cells were grown adherently in RPMI medium with FCS (under “differentiation” conditions), ALDH^+^ cells tended to reconstruct ALDH^−^ subpopulation, but not *vice versa* (Figure 2C).

### ALDH^+^ and ALDH^−^ cells are equally tumourigenic *in vivo*

**Figure 3:**
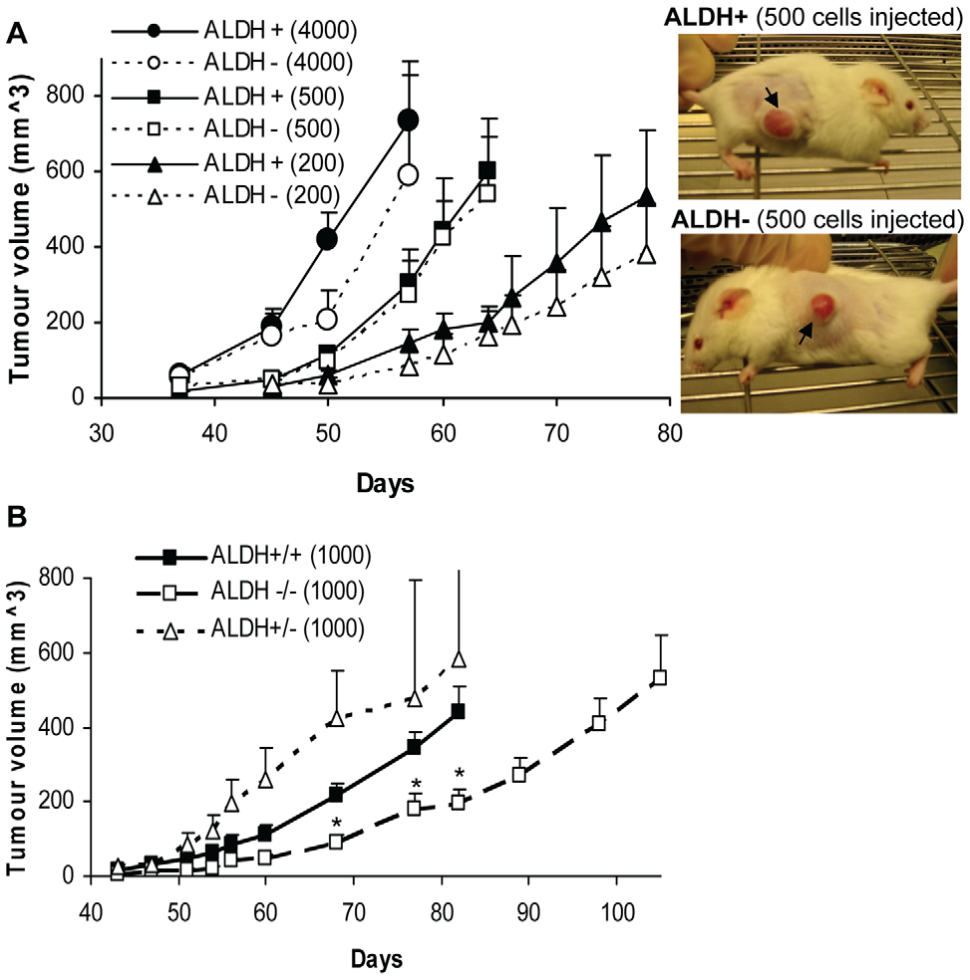
*In vivo* tumour growth initiated by ALDH^+^ and ALDH^−^ cells isolated from Melmet 1 xenografts. (A) Sorted cells from both subpopulations were injected s.c. (4000, 500 and 200 cells per injection, indicated in the figure) into NOD-SCID Il2rg^−/−^ mice for formation of 1^st^ generation tumours (appearance of the tumours is shown in the photographs). P>0.05 at all time points in the comparable groups. (B) The tumours derived from ALDH^+^ and ALDH^−^ cells were resorted into ALDH^+/+^, ALDH^+/−^ and ALDH^−/−^ subpopulations (see Figure 4), and 1000 viable cells from each subpopulation were reinjected into mice for formation of 2^nd^ generation tumours. Data represent mean ± SEM of 4–6 tumours. P>0.05 between ALDH^+/+^ and ALDH^+/−^; p<0.05 between ALDH^+/+^ and ALDH^−/−^ indicated by *.

ALDH^+^ and ALDH^−^ subpopulations were isolated from different xenografts and two patient biopsies, and the tumourigenic potential of the isolated cells was compared *in vivo* by s.c. injection into NOD-SCID Il2rg^−/−^ mice. As shown in Table 1 and illustrated in Figure 3A, both ALDH^+^ and ALDH^−^ cells derived from the xenografts were capable of efficient formation of 1^st^ generation tumours following injection of as few as 200 cells. Also, the two subpopulations isolated from patient biopsies did not show significant differences with respect to tumourigenic abilities: 1000 cells from ALDH^+^ and ALDH^−^ formed tumours at 7 of 12 (58%) and 6 of 11 (55%) sites, respectively; 500 cells failed to induce tumours (0/4) in both cases during 6 months *in vivo*.

**Table 1:** Efficiency of tumour formation (# tumours/# injections (%)).

Subpopulation	# Cells injected					**Transplanted
	10,000	4,000	1,000	500	200	
**ALDH^+^**	6/6 (100%)	4/4 (100%)		6/6 (100%)	4/4 (100%)	6/6 (100%)
**ALDH^−^**	7/7 (100%)	4/4 (100%)		6/6 (100%)	4/4 (100%)	8/8 (100%)
* **ALDH^+/+^**			5/6 (83%)			
* **ALDH^+/−^**			4/6 (67%)			
* **ALDH^−/−^**			5/6 (83%)			

*The harvested 1^st^ generation tumours formed from sorted ALDH^+^ or ALDH^−^ cells (the tumour growth curves are shown in Figure 3A) were FACS-separated into positive and negative subpopulations, which were reinjected into mice for studies of the formation of 2^nd^ generation tumours (the tumour growth curves are shown in Figure 3B).

**The 1^st^ generation tumours formed from the injected sorted ALDH^+^ and ALDH^−^ cells were transplanted into new mice for evaluation of the formation of 2^nd^ generation tumours.

Further characterization of 1^st^ generation tumours induced by the ALDH^+^ and ALDH^−^ cells derived from the xenografts, revealed that the tumour growth rate was not dependent (p>0.05) on the ALDH phenotype (Figure 3A). Besides, it was shown that the 1^st^ generation tumours derived either from ALDH^+^ or ALDH^−^ cells can be serially passaged *in vivo*. Thus, following transplantation, 2^nd^ generation tumours were generated with 100% efficiency in both cases (Table 1). Furthermore, ALDH^−^ cells FACS-isolated from ALDH^−^ derived tumours (designated as ALDH^−/−^) could form 2^nd^ generation tumours with the same efficiency and similar lag-time (40–47days) before palpability, as ALDH^+^ cells isolated from ALDH^+^ derived tumours (designated as ALDH^+/+^) (Table 1). It should be noted that ALDH^−/−^ tumours demonstrated slower growth than ALDH^+/+^ (p<0.05 at weeks ∼10–12), whereas there was no statistically significant differences (p>0.05) between ALDH^+/−^ and ALDH^+/+^ tumour growth (Figure 3B). The latter observation suggests that tumours induced by the ALDH negative subpopulations do not consistently show slower growth than the tumours initiated by the positive counterparts. Most important, Figure 3B reveals that the ALDH^−/−^ tumours also demonstrate unlimited growth, i.e. they reach a diameter>10 mm, although with 2.5-weeks delay compared to the ALDH^+/+^ tumours.

**Figure 4:**
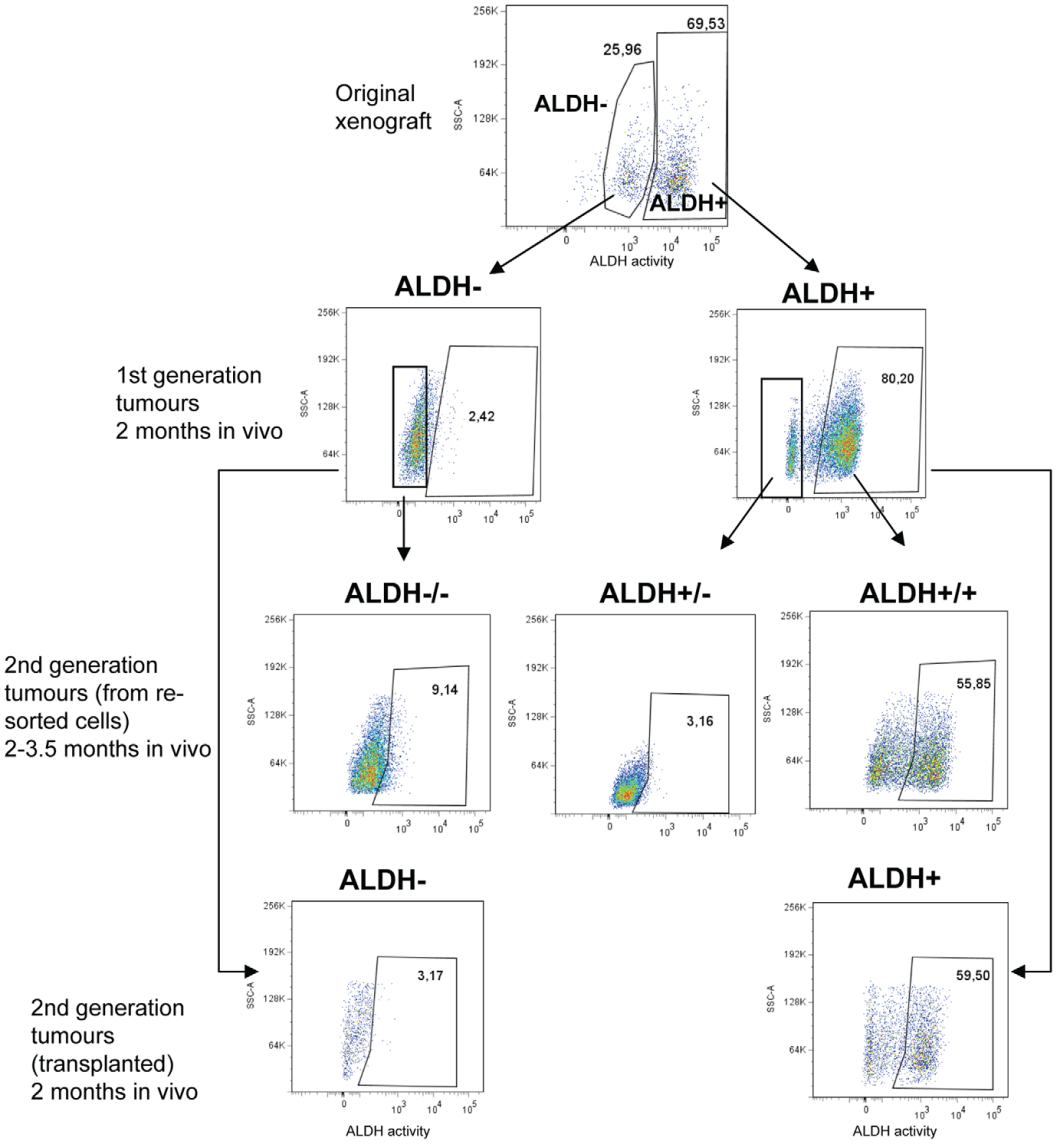
Identification of ALDH^+^ and ALDH^−^ subpopulations in 1^st^ and 2^nd^ generation tumours. Sorted viable ALDH^+^ and ALDH^−^ cells from the Melmet 1 xenograft were injected s.c (the tumour growth curves are presented in Figure 3A), and the formed 1^st^ generation tumour were: i) resorted into ALDH^+/+^, ALDH^+/−^, ALDH^−/−^ subpopulations that were reinjected for formation of 2^nd^ generation tumours (the growth curves are presented in Figure 3B); ii) divided into small peaces of tumour tissue that were transplanted for formation of 2^nd^ generation tumours. All formed tumours were reanalyzed by the Aldefluor assay. The gates were set based on “+DEAB controls” (not shown).

To compare the capacity of the ALDH^+^ and ALDH^−^ cells to reestablish tumour heterogeneity, the 1^st^ and 2^nd^ generation tumours were harvested and reanalyzed by the Aldefluor assay to elucidate their ALDH phenotype. The representative data is shown in Figure 4. As can be seen, the tumours derived from ALDH^−^ cells basically kept the ALDH^−^ phenotype (Figure 4, left/middle branch, n = 7), corresponding to the data *in vitro* (Figure 2B,C). In contrast, ALDH^+^ derived tumours had a mixed phenotype, where 20–40% of the melanoma cells did not show ALDH activity, whereas remaining 60–80% staid ALDH positive (Figure 4, right branch, n = 7) This confirms higher abilities of the ALDH^+^ cells to reestablish tumour heterogeneity, i.e. the existence of “cellular hierarchy” with respect to ALDH.

### ALDH phenotype and response to therapy

**Figure 5:**
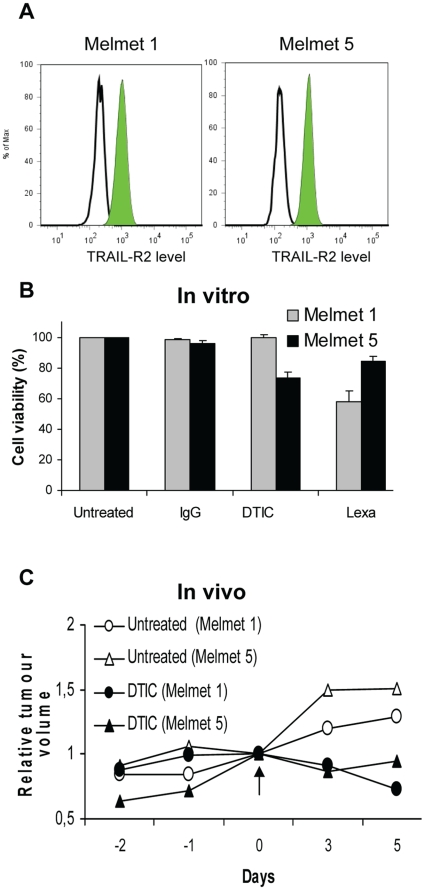
Evaluation of TRAIL-R2 levels and treatment effects *in vitro* and *in vivo*. (A) TRAIL-R2 levels. Solid lines, isotype-matched controls; shaded areas, TRAIL-R2. (B) Cell viability (detected by the MTS assay) following *in vitro* treatment with 100 µg/ml DTIC or 10 µg/ml lexatumumab (Lexa) for two days. Results are expressed as median cell survival relative to untreated control cells, and bars denote SEM from 2–6 independent experiments (each performed in triplicate). (C) Relative tumour volume (normalized to the volume at day 0) in control and DTIC-treated mice. DTIC (250 mg/kg) was injected i.p. at day 0 (indicated by an arrow), and the tumours were harvested at days 3 and 5 for detection of ALDH^+^ subpopulations by the Aldefluor assay (as shown in Figure 6B). Data represent average volume (n = 4 and n = 8 for controls and treated groups, respectively).

To evaluate whether ALDH^+^ subpopulation is associated with drug resistance, the response of ALDH^+^ and ALDH^−^ cells to two different drugs was compared. The approved drug, an alkylating agent dacarbazine (DTIC), and the experimental drug lexatumumab were used. Lexatumumab is a fully human agonistic antibody that specifically binds the death receptor TRAIL-R2, activating the extrinsic apoptotic pathway [20]. Melmet 1 and Melmet 5 cells in culture uniformly express TRAIL-R2 (Figure 5A), validating these cell lines as potential targets of lexatumumab. The short-term cytotoxic effect of the drugs is show in Figure 5B, revealing that Melmet 5 responded moderately to treatment with DTIC, while Melmet 1 was resistant; however, Melmet 1 showed higher response to lexatumumab than Melmet 5.

**Figure 6:**
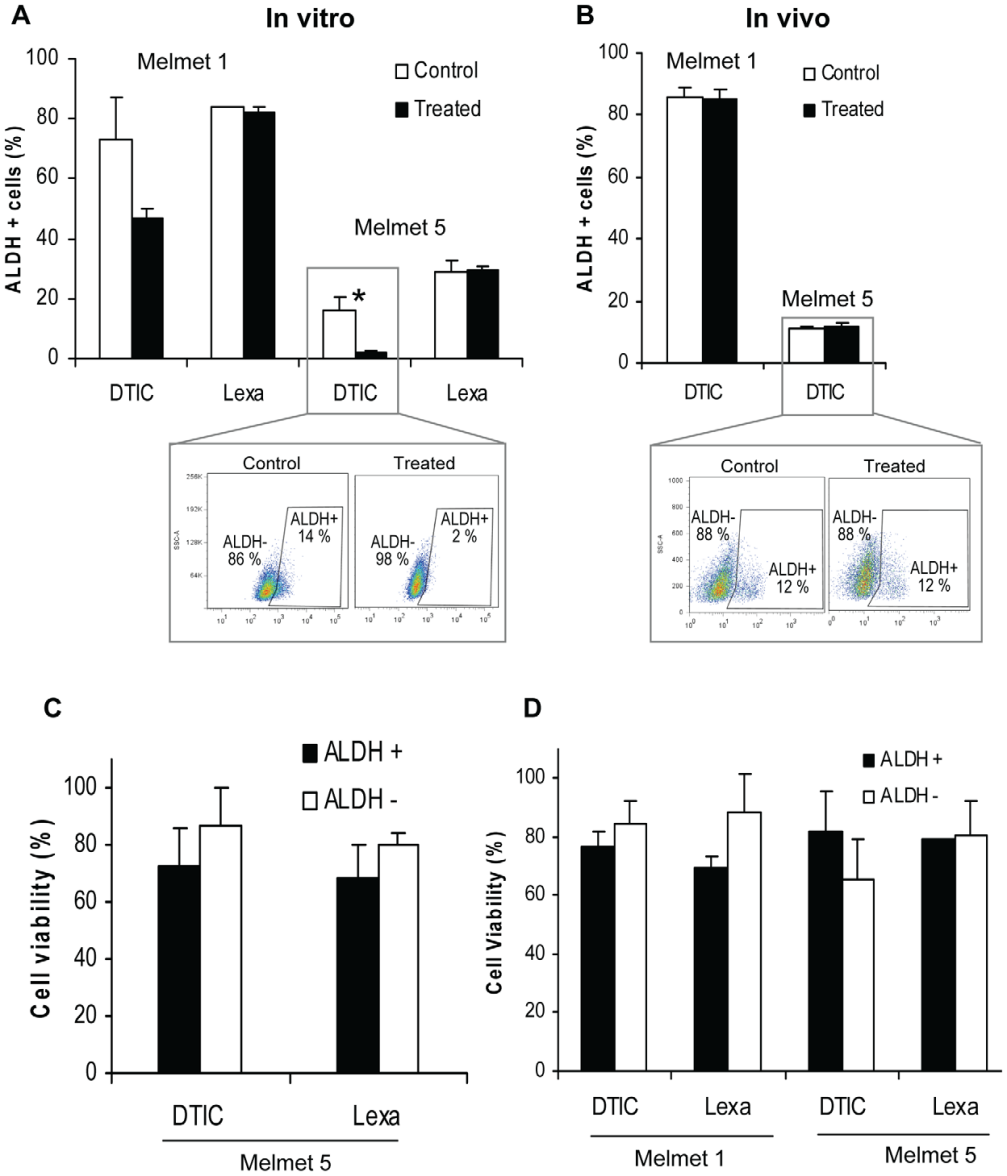
Comparison of ALDH^+^ and ALDH^−^ cells, derived from Melmet cultures (A, C) and xenografts (B, D), with regard to response to treatment. (A) *In vitro*: the percentage of ALDH^+^ and ALDH^−^ cells in the control samples (untreated or IgG-treated, respectively) and samples treated with 100 µg/ml DTIC or 10 µg/ml lexatumumab (Lexa) for two days, was determined by the Aldefluor assay as illustrated in the insert and Figure S5A. The graphs show the treatment effect on the percentage of ALDH^+^ cells only; error bars indicate SEM from 2–3 experiments. *, p<0.05. (B) Melmet tumours were treated *in vivo* by injecting 250 mg/kg DTIC i.p. (effects on tumour growth is shown in Figure 5C), and the percentage of ALDH^+^ and ALDH^−^ cells in control and treated tumours at day 3 (and day 5, not shown) was evaluated by the Aldefluor assay as illustrated in the insert and Figure S5B. The percentages of ALDH^+^ cells±SEM from 2–3 tumours are presented. (C, D) The ALDH^+^ and ALDH^−^ cells were FACS-sorted from the culture (C) and the xenografts (D) and treated *in vitro* with DTIC or lexatumumab for two days before the MTS assay. Results are expressed as median cell survival relative to untreated cells, and bars denote SD (single experiment performed in triplicate) (C) and SEM from 2–3 independent experiments (D); p>0.05 in all compared groups, ALDH^+^ versus ALDH^−^.

To investigate whether ALDH^+^ and ALDH^−^ subpopulations are differentially affected by the drugs, the percentage of each subpopulation before and after the treatment was estimated as indicated in Figure S5. Relative enrichment of one of the subpopulation as a consequence of the treatment would indicate enhanced treatment-resistance in this subpopulation. Previous studies in other cancers indicated an association between drug-resistance and ALDH^+^ cells [11], [12], [21], therefore, the effect of DTIC and lexatumumab primarily on the percentage of ALDH^+^ cells was evaluated as shown in Figure 6 A, B. As can be seen, no changes in the proportion of ALDH^+^ cells were observed following *in vitro* treatment with lexatumumab, whereas DTIC reduced the percentage of ALDH^+^ cells, consequently, increased the percentage of ALDH^−^ cells (Figures 6A and S5A). Overnight-chase in DTIC-free medium before the Aldefluor assay did not restore the proportion of ALDH^+^ cells (data not shown), implying an irreversible effect on ALDH^+^ subpopulation. However, *in vivo* DTIC treatment of mice bearing Melmet tumours did not suggest an elevated sensitivity of the ALDH^+^ cells. Thus, the percentage of ALDH^+^ cells was not changed in the treated tumours compared to untreated controls (Figures 6B and S5B), even though inhibitory effect of DTIC on tumour growth/volume was documented (Figure 5C).

To investigate the treatment effect selectively on ALDH^+^ and ALDH^−^ cells, the two distinct subpopulations were FACS-isolated from cell cultures and xenografts and exposed to the drugs *in vitro*. The short-term and long-term effects of the treatment were evaluated by the MTS and clonogenic assays, respectively. Both subpopulations, isolated either from the cell culture (here we analyzed only Melmet 5) (Figure 6C) or from different xenografts (Figure 6D), demonstrated similar (p>0.05) viability when evaluated by the MTS assay. Likewise, the clonogenicity of both subpopulations appeared to be similar when the sorted cells were cultured for two weeks in the presence of DTIC or lexatumumab. After DTIC treatment, both subpopulations were capable forming only small “colonies” harbouring less-than-50 cells, while lexatumumab treatment completely blocked colony formation in both subpopulations (data not shown). This indicates that clonogenicity of both, ALDH^+^ and ALDH^−^ cells was strongly inhibited by the prolonged treatments.

Summarising, neither of the two investigated drugs showed higher cytotoxic effect on ALDH^−^ compared to ALDH^+^ cells, suggesting that in examined malignant melanoma model systems, the drug-response is not dependent on the ALDH^+^ phenotype. Seemingly higher sensitivity of ALDH^+^ cells to DTIC *in vitro*, as detected by Aldefluor assay (Figure 6A), was not confirmed *in vivo* (Figure 6B) or on sorted cells (Figure 6C, D), where similar response of both subpopulations was observed.

## Discussion

So far, there is no definite evidence that malignant melanoma follows a CSC model, and there are no proved markers that would distinguish tumourigenic from non-tumourigenic melanoma cells [7]. However, this does not exclude a possibility that such markers might exist, and if they do, they are expected to “mark” a large subpopulation of cells, since clonogenic/tumourigenic melanoma cells seem to exist at high frequencies [7], [8]. Furthermore, there is a possibility that markers might identify cell subpopulations showing differences with respect to biological aggressiveness, including therapeutic resistance. Identification of cell subsets that demonstrate enhanced resistance to drugs would be of importance for melanoma therapy. Also, it might help to uncover molecular features associated with melanoma resistance.

Although, ALDH is not recognized as a generic marker of stem cells [22], it appeared to be a valuable functional marker for isolation of cells with tumour-initiating, metastatic and drug-resistance properties in cancers that follow a CSC model, like leukaemia [23] and breast cancer [9], [10]. We have shown that in examined melanoma models, a relatively large subpopulation of cells had elevated activity of ALDH. Furthermore, ALDH activity in melanoma patient biopsies seemed to correlate to expression of the cell surface proteoglycan, melanoma associated antigen HMW-MAA. HMW-MAA was shown to stimulate melanoma cell growth, migration and epithelial-mesenchymal transition promoting tumour progression [19]. Therefore, we reasoned that ALDH might be a potentially interesting marker for identification of melanoma cells with enhanced biological aggressiveness. However, our present data indicate that there is no correlation between ALDH activity and the clonogenic/tumourigenic capacity, or enhanced drug-resistance, providing additional evidence that ALDH is not a universal marker of aggressive tumourigenic cells. Although melanomas showed heterogeneity with regard to ALDH, both ALDH^+^ and ALDH^−^ cells could self-renew, form clones *in vitro* and generate tumours that could be passaged *in vivo*. The clonal spheroids generated from sorted single cells and grown in stem cell medium, mainly retained the parental ALDH phenotype, i.e. either ALDH^+^ or ALDH^−^ (Figure 2B), confirming that: i) cell sorting definitely separated two distinct subpopulations; ii) both subpopulations were intrinsically clonogenic. Also tumours derived from ALDH^−^ cells basically kept a parental ALDH^−^ phenotype during two-three passages *in vivo* (Figure 4). This further confirms that the observed tumourigenicity is a characteristic of ALDH^−^ cells, and not a result of poor separation of the two subpopulations. In contrast, the tumours derived from ALDH^+^ cells consisted of mixed subpopulations–a majority of the cells were ALDH^+^, whereas 20–40% did not show ALDH activity. Altogether, this suggests that ALDH^+^ cells have higher abilities to reestablish tumour heterogeneity, at least with respect to ALDH. There could be several reasons for this. If melanoma was hierarchically organized and followed a CSC model, one could speculate that ALDH^+^ cells localize higher in the cellular hierarchy, have stem cell characteristics and, therefore, can recapitulate the phenotypic heterogeneity, which was not possible for ALDH^−^ cells. However, no other evidence indicating that ALDH^+^ cells are more CSC-like and thus more tumourigenic compared to ALDH^−^ were found. Therefore, the simplest interpretation would be that some ALDH^+^ cells lose the ALDH activity under the influence of the *in vivo* microenvironment. Since this activity does not seem to be crucial for tumour-formation, both subpopulations could eventually contribute to the tumour growth. It should be noted that recently Held et al. showed using mouse melanoma cells, that the ability to reestablish heterogeneity (in this case, with respect to CD34^+/−^ p75^+/−^ phenotype) not necessarily correlates to the enhanced capacity of tumour/colony-initiation [24], which is in agreement with our data.

Enhanced resistance to therapy is another characteristic of ALDH^+^ cells identified in cancers like breast and colorectal cancers that seem to follow a CSC model [11], [12], [21]. In contrast, no correlation between ALDH^+^ subpopulation and therapeutic resistance was observed in the examined melanoma models. It should be mentioned that, unlike the classical chemotherapeutic agent cyclophosphamide (a target of the ALDH1A1 enzymatic activity), the drugs that we used (DTIC and lexatumumab) are not substrates of ALDH. However, previous studies have reported a correlation between ALDH^+^ cells and enhanced resistance to drugs that are not substrates of ALDH [11], [12], [21], indicating that the therapeutic resistance was mediated by mechanism not related to the ALDH enzymatic activity, but somehow activated in ALDH^+^ subpopulations. It has been speculated that these mechanisms are associated with stemcellness of ALDH^+^ cells [12], [21]. In highly aggressive melanoma, however, such mechanisms do not seem to be restricted to ALDH^+^ cells, since these cells did not demonstrate higher resistance than ALDH^−^ cells to the investigated drugs. In contrast to the situations in other cancers, [11], [12], [21], a reduction of the ALDH^+^ fraction was observed following DTIC treatment *in vitro* (Figure 6A), implying that ALDH^+^ cells might be more sensitive to DTIC. This, however, was not confirmed *in vivo* (Figure 6B). Since none of our other assays, or *in vivo* studies (Figure 6 B–D) confirmed that DTIC preferentially “targets” ALDH^+^ subpopulation, we assume that both, ALDH^+^ and ALDH^−^ cells respond to DTIC and lexatumumab similarly. The *in vitro* observed reduction of the ALDH^+^ proportion we explain by the DTIC-induced interference with the Aldefluor assay, e.g. inhibition of the ALDH enzymatic activity when the treatment was performed *in vitro*. Thus, the therapy-related data further argues against ALDH as a marker of melanoma cells with enhanced aggressive properties.

To conclude, malignant melanoma harbours a large fraction of cells positive for ALDH. However, melanoma cells lacking ALDH activity (ALDH^−^) are equally resistant to treatment, equally tumourigenic and can be serially transplanted *in vivo* like cells with ALDH activity (ALDH^+^). This indicates that in highly aggressive melanoma, the functional “marker” ALDH does not discriminate cells with enhanced biological aggressiveness, and ALDH^+^ subpopulation does not play an exclusive role in tumour initiation and/or in low response to therapy. Although, ALDH^+^ melanoma cells show higher abilities for generating phenotypic heterogeneity, the implication of this remains unknown, and the present data suggests that it is not associated with clonogenic and tumourigenic differences nor with differences in drug-resistance. It should be added that it has not been investigated whether ALDH activity could be more discriminatory in less aggressive tumours or primary melanomas. Here presented data, based on the melanoma models and the patient biopsies that represent very aggressive advanced-stage disease, can not exclude such possibility.

## Supporting Information

Figure S1 Gating strategy for flow cytometry analysis of Aldefluor-stained samples. Cells derived from disintegrated *in vivo* samples were stained by the Aldefluor assay, followed by staining with human specific anti-TRA-1-85. All samples were stained with PI just before flow cytometry. All samples were analyzed by sequential gating including main population (G1), single cells (G2), viable (PI negative) cells (G3), TRA-1-85-positive (i.e. human) cells (G4). Controls with DEAB inhibitor (“+DEAB”) were used to set a gate G5, which helps to identify the ALDH^+^ subpopulation in the test samples without DEAB inhibitor (“−DEAB”).
(0.67 MB TIF)

Figure S2 Identification of ALDH^+^ subpopulations in various melanoma models *in vitro* and *in vivo*. Representative dot-plots for Melmet 1 (A) and Melmet 5 (B) monolayer cultures (MON), spheroid cultures (SPH) and xenografts derived from the corresponding MON and SPH (A, B). (C) Identification of the ALDH^+^ subpopulation in the xenograft established from directly implanted patient LN biopsy. “+DEAB controls” were used to set the gates defining ALDH^+^ populations in “−DEAB” samples.
(0.84 MB TIF)

Figure S3 Analysis of LN biopsies #129 and #135 (see also Figure 1A) with respect to HMW-MAA expression and ALDH activity. FACS analysis was performed following the strategy described in Figure S1. The presence of melanoma cells in the biopsies as well as in the sorted HMW-MAApositive, but not in HMW-MAAnegative fractions, was confirmed by Melan A staining (using the antibody clone A103 (Dako) at dilution 1∶20; DAPI for nuclear counterstain). Melan A staining-green, DAPI -blue. HMW-MAA expression was identified by staining with antibody 9.2.27-APC; median of fluorescence (Fl.) intensity in HMW-MAA+ subpopulation is indicated in the histograms. ALDH activity in HMW-MAApositive and HMW-MAAnegative (where possible) fractions was analyzed by the Aldefluor assay.
(3.53 MB TIF)

Figure S4 Analysis of the additional LN biopsies #127 and #126 representing high and low expression of HMW-MAA, respectively. The samples were analyzed as described in Figure S3. The presence of melanoma cells in the biopsy was confirmed by Melan A staining. Median of fluorescence (Fl.) intensity in HMW-MAA^+^ subpopulation is indicated in the histograms. ALDH activity in HMW-MAApositive and HMW-MAAnegative (where possible) fractions was analyzed by the Aldefluor assay.
(1.74 MB TIF)

Figure S5 Treatment effect on ALDH^+^ and ALDH^−^ cells derived from cultures (A) and xenografts (B). The treatment was performed as described in Figure 6A, B, and the viable cells were subjected to the Aldefluor assay. The gates were defined based on “+DEAB controls”, and the ALDH^+^ and ALDH^−^ subpopulations were identified in “−DEAB test samples”. The percentages of ALDH^+^ and ALDH^−^ cells are indicated in blue and black, respectively.
(1.72 MB TIF)
