# Supplementary figures and images for: Aldehyde Dehydrogenase (ALDH) Activity Does Not Select for Cells with Enhanced Aggressive Properties in Malignant Melanoma
Source: PLoS One. 2010 May 20;5(5):e10731. doi: 10.1371/journal.pone.0010731 (PMC2874003; doi:10.1371/journal.pone.0010731)

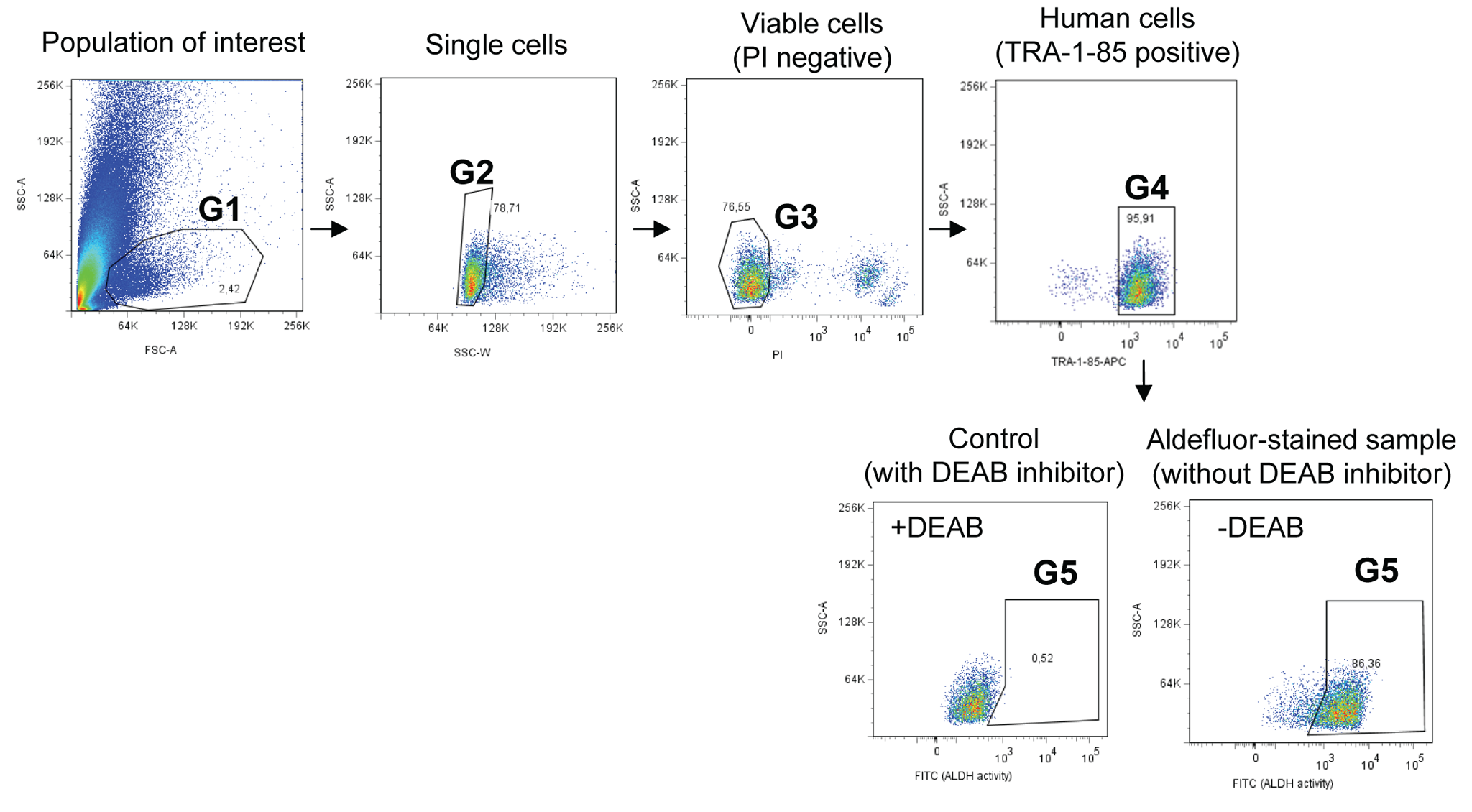

Supplement: Figure S1 — Gating strategy for flow cytometry analysis of Aldefluor-stained samples. Cells derived from disintegrated in vivo samples were stained by the Aldefluor assay, followed by staining with human specific anti-TRA-1-85. All samples were stained with PI just before flow cytometry. All samples were analyzed by sequential gating including main population (G1), single cells (G2), viable (PI negative) cells (G3), TRA-1-85-positive (i.e. human) cells (G4). Controls with DEAB inhibitor (“+DEAB”) were used to set a gate G5, which helps to identify the ALDH+ subpopulation in the test samples without DEAB inhibitor (“−DEAB”). (0.67 MB TIF) [file pone.0010731.s001.tif]

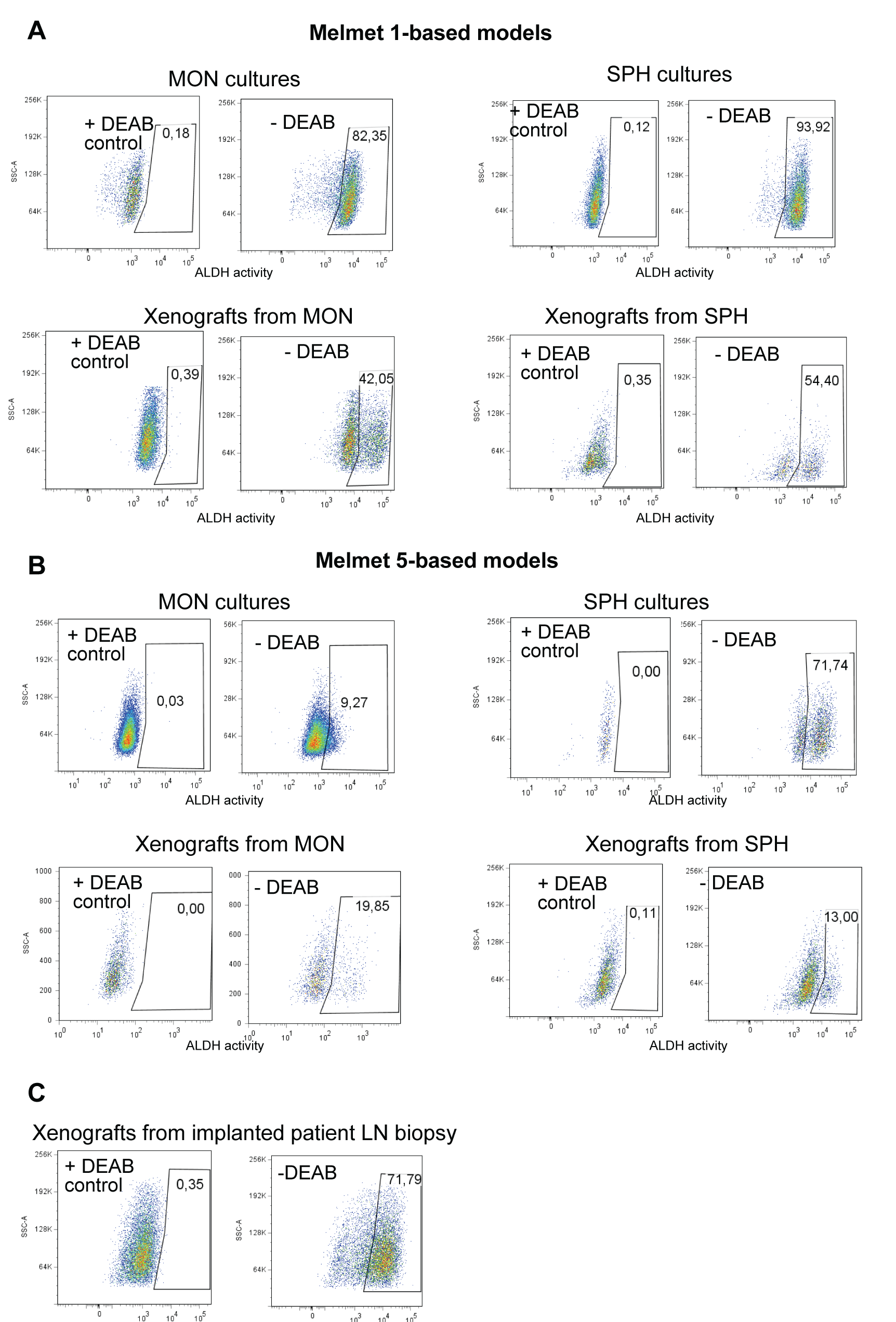

Supplement: Figure S2 — Identification of ALDH+ subpopulations in various melanoma models in vitro and in vivo. Representative dot-plots for Melmet 1 (A) and Melmet 5 (B) monolayer cultures (MON), spheroid cultures (SPH) and xenografts derived from the corresponding MON and SPH (A, B). (C) Identification of the ALDH+ subpopulation in the xenograft established from directly implanted patient LN biopsy. “+DEAB controls” were used to set the gates defining ALDH+ populations in “−DEAB” samples. (0.84 MB TIF) [file pone.0010731.s002.tif]

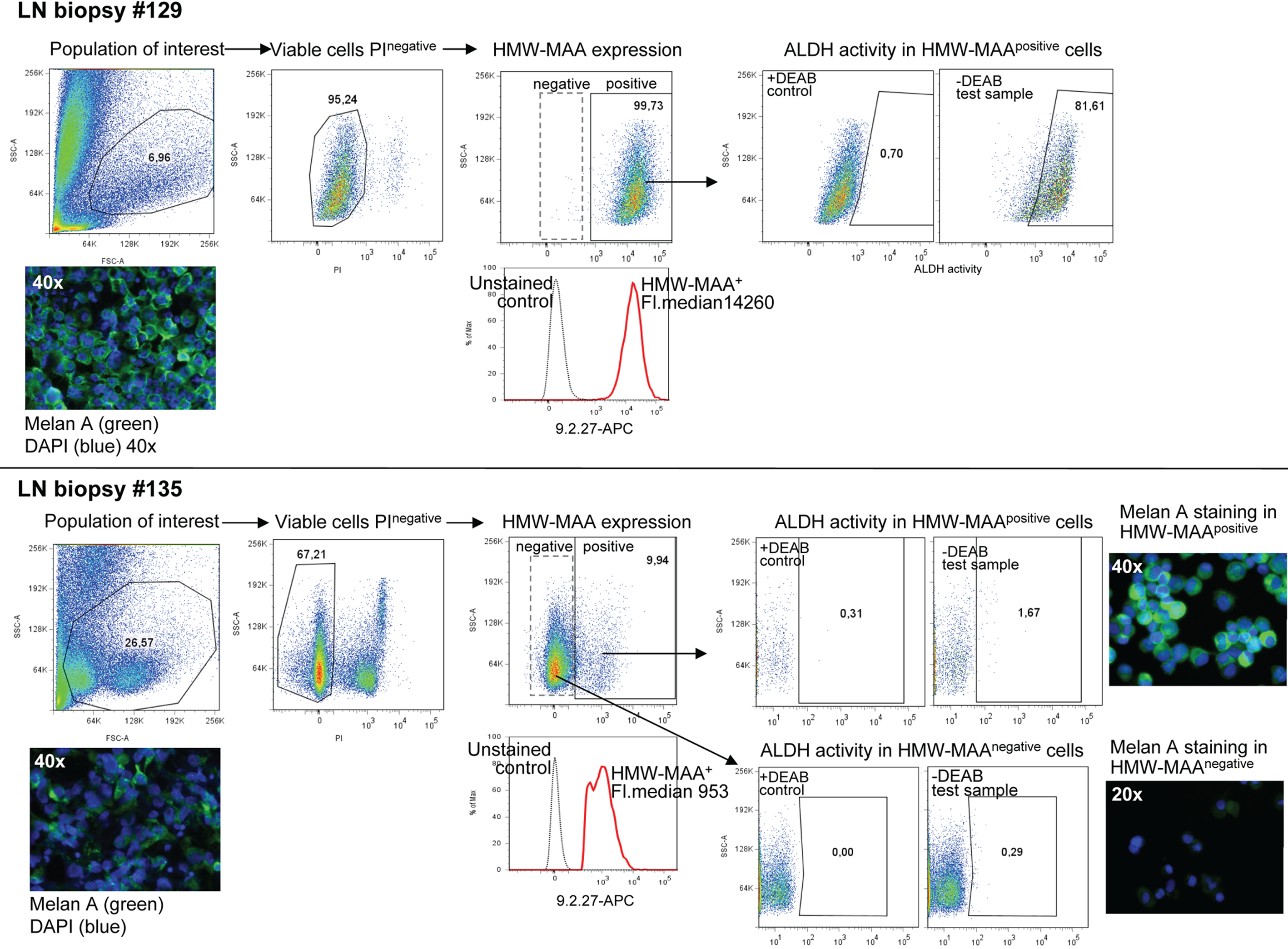

Supplement: Figure S3 — Analysis of LN biopsies #129 and #135 (see also Figure 1A) with respect to HMW-MAA expression and ALDH activity. FACS analysis was performed following the strategy described in Figure S1. The presence of melanoma cells in the biopsies as well as in the sorted HMW-MAApositive, but not in HMW-MAAnegative fractions, was confirmed by Melan A staining (using the antibody clone A103 (Dako) at dilution 1∶20; DAPI for nuclear counterstain). Melan A staining-green, DAPI -blue. HMW-MAA expression was identified by staining with antibody 9.2.27-APC; median of fluorescence (Fl.) intensity in HMW-MAA+ subpopulation is indicated in the histograms. ALDH activity in HMW-MAApositive and HMW-MAAnegative (where possible) fractions was analyzed by the Aldefluor assay. (3.53 MB TIF) [file pone.0010731.s003.tif]

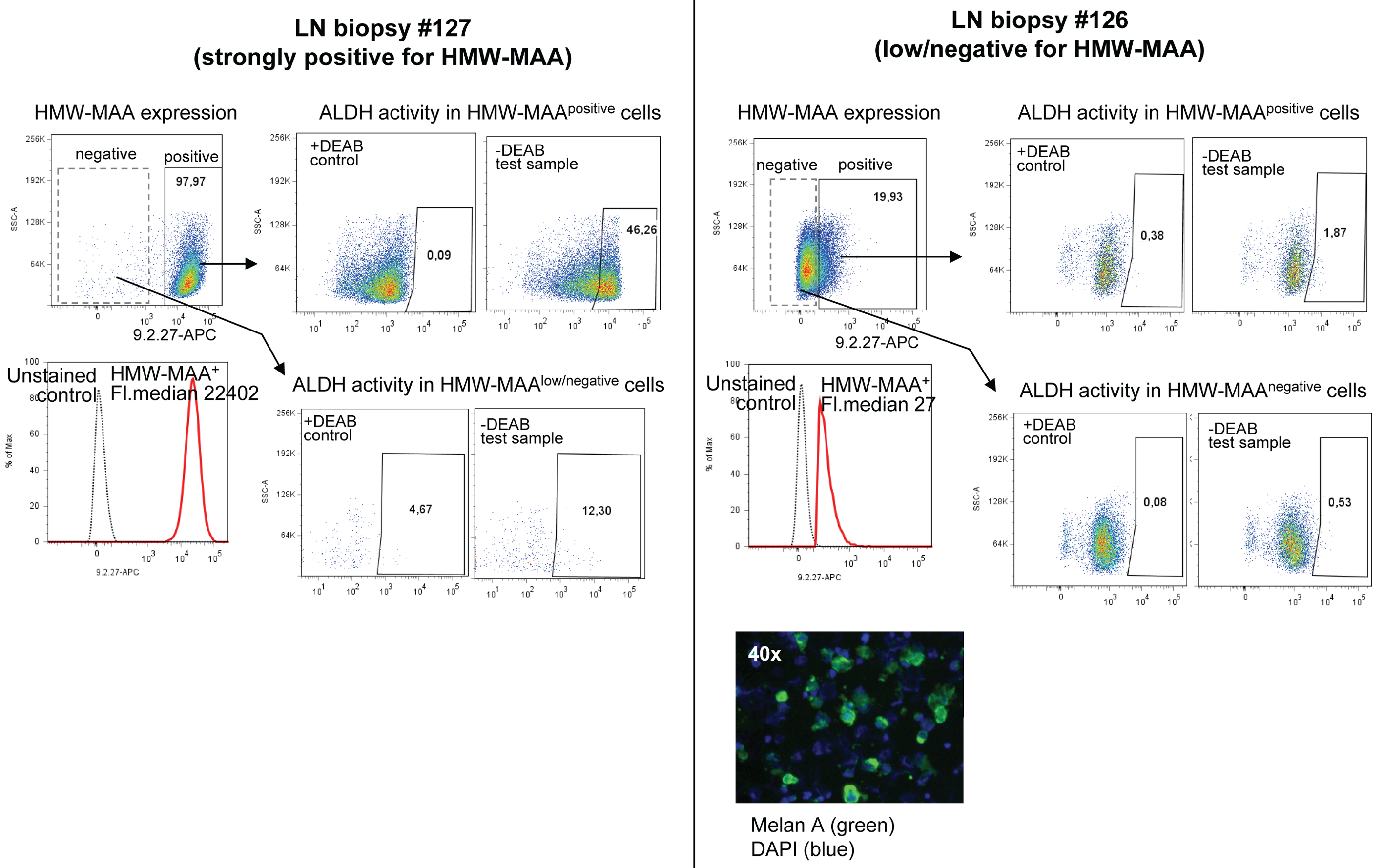

Supplement: Figure S4 — Analysis of the additional LN biopsies #127 and #126 representing high and low expression of HMW-MAA, respectively. The samples were analyzed as described in Figure S3. The presence of melanoma cells in the biopsy was confirmed by Melan A staining. Median of fluorescence (Fl.) intensity in HMW-MAA+ subpopulation is indicated in the histograms. ALDH activity in HMW-MAApositive and HMW-MAAnegative (where possible) fractions was analyzed by the Aldefluor assay. (1.74 MB TIF) [file pone.0010731.s004.tif]

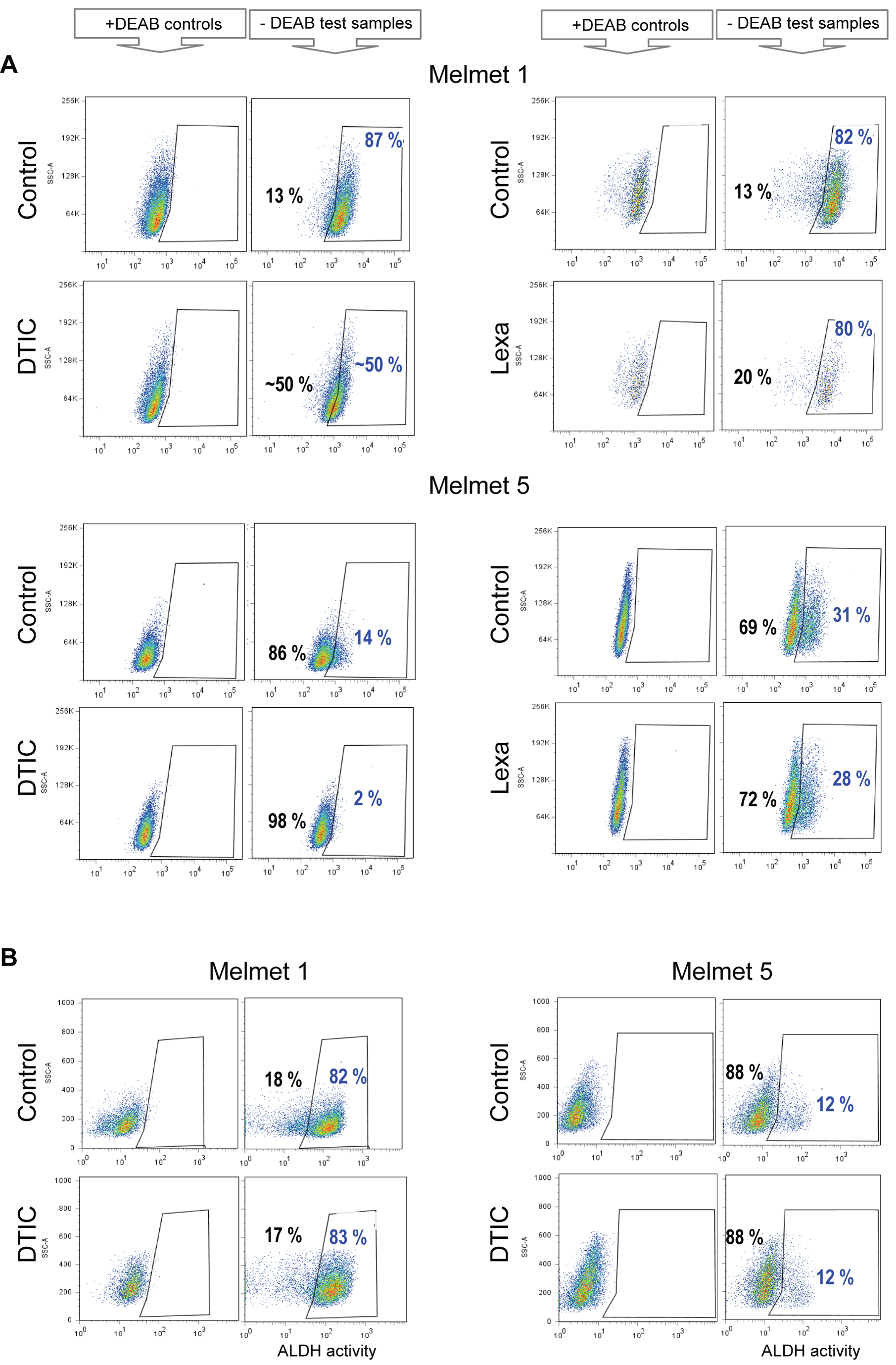

Supplement: Figure S5 — Treatment effect on ALDH+ and ALDH− cells derived from cultures (A) and xenografts (B). The treatment was performed as described in Figure 6A, B, and the viable cells were subjected to the Aldefluor assay. The gates were defined based on “+DEAB controls”, and the ALDH+ and ALDH− subpopulations were identified in “−DEAB test samples”. The percentages of ALDH+ and ALDH− cells are indicated in blue and black, respectively. (1.72 MB TIF) [file pone.0010731.s005.tif]
